# Prognostic Value and Therapeutic Perspectives of Coronary CT Angiography: A Literature Review

**DOI:** 10.1155/2018/6528238

**Published:** 2018-09-16

**Authors:** Patrizia Carità, Andrea Igoren Guaricci, Giuseppe Muscogiuri, Nazario Carrabba, Gianluca Pontone

**Affiliations:** ^1^Department of Cardiology, Santissima Trinita' Hospital, Borgomanero, Novara, Italy; ^2^Cardiology Institute, Department of Emergency and Organ Transplantation, University Hospital Policlinico Consorziale of Bari, Bari, Italy; ^3^Centro Cardiologico Monzino, IRCCS, Milan, Italy; ^4^Division of Cardiology, Careggi Hospital, Florence, Italy

## Abstract

Coronary stenosis severity is both a powerful and a still debated predictor of prognosis in coronary artery disease. Coronary computed tomographic angiography (CCTA) has emerged as a noninvasive technique that enables anatomic visualization of coronary artery disease (CAD). CCTA with newer applications, plaque characterization and physiologic/functional evaluation, allows a comprehensive diagnostic and prognostic assessment of otherwise low-intermediate subjects for primary prevention. CCTA measures the overall plaque burden, differentiates plaque subtypes, and identifies high-risk plaque with good reproducibility. Research in this field may also advance towards an era of personalized risk prediction and individualized medical therapy. It has been demonstrated that statins may delay plaque progression and change some plaque features. The potential effects on plaque modifications induced by other medical therapies have also been investigated. Although it is not currently possible to recommend routinely serial scans to monitor the therapeutic efficacy of medical interventions, the plaque modulation, as a part of risk modification, appears a feasible strategy. In this review we summarize the current evidence regarding vulnerable plaque and effects of lipid lowering therapy on morphological features of CAD. We also discuss the potential ability of CCTA to characterize coronary atherosclerosis, stratify prognosis of asymptomatic subjects, and guide medical therapy.

## 1. Introduction

The diagnostic approach to cardiac and coronary diseases is rapidly changing with the advent and implementation of radiologic techniques [1–14]. Coronary computed tomographic angiography (CCTA) is increasingly emerging as a noninvasive technique that enables direct anatomic visualization of atherosclerotic stenosis in the epicardial coronary arteries, with low radiation exposure [15–18]. Although such factors (i.e., high heart rate, arrhythmia, obesity, and high coronary calcium burden) may limit overall evaluability [19–21], the significant improvement in technologies during the last past decades has opened new perspectives in cardiac imaging permitting the acquisition within few seconds and with a higher spatial resolution [22–24]. CCTA has proven to have a high diagnostic accuracy compared with the invasive coronary angiography (ICA), which represents until now the standard of reference for evaluating coronary artery disease [25–33]. Using at least a 64-slice multidetector row, a sensitivity and specificity of 98% and 90%, respectively, have been reported on a per patient level. The elevated sensitivity turns out into a negative predictive value (NPV) ranging from 95 to 100% to rule out obstructive coronary artery disease (CAD) [23]. This high negative predictive value for CAD translates into an excellent negative predictive value for future events. In a recent study analyzing more than six hundred patients, normal CCTA findings were associated with an event-free survival rate of 100% in both diabetic and nondiabetic patients with suspected CAD [34].

In 2013, the European Society of Cardiology proposed CCTA as an alternative to stress imaging techniques for the assessment of patients with suspected stable CAD and low-to-intermediate pretest probability of CAD [35]. Recently, the update of the NICE-UK guidelines on the management of patients with new onset chest pain proposed CCTA as first-line diagnostic tool for people in whom stable angina cannot be excluded by clinical assessment alone [36].

In this context, coronary stenosis severity is considered a powerful although debated prognostic index of CAD prognosis. Both invasive and noninvasive angiographic studies have demonstrated the correlation between stenosis degree and clinical events. However, in a recent study Min et al. evaluated a large consecutive cohort of patients without history of CAD and showed a similar incidence of all-cause mortality in nonobstructive and 1-vessel obstructive CAD as assessed by CCTA (HR: 1.62 vs. 1.75) [37]. Moreover, it has been reported that more than two-thirds of acute myocardial infarction (MI) may be due to nonobstructing lesions [38]. Beyond the degree of stenosis, other features are pivotal determinants of events. Numerous clinical biomarkers and imaging modalities have been investigated during the past few decades in order to identify patients harboring plaques at high risk of rupturing (vulnerable plaque), hoping to be able to prognosticate events. While ICA is focused only on the evaluation of the degree of coronary stenosis (luminography), CCTA looking at both the wall and the lumen of coronary artery reliably measures the overall plaque burden, differentiates plaque subtypes, and identifies adverse features of coronary high-risk plaques [39, 40]. In addition, CCTA may help us to avoid a PCI in case of obstructive CAD in a small vessel and may help us to start an early and aggressive optimal medical therapy in case of nonobstructive extensive CAD. Currently, there are increasing interest and continuing debate on the potential role of CCTA as a noninvasive method for mapping CAD, identifying nonobstructive lesions with features of vulnerability, defining prognosis of otherwise low-to-moderate risk subjects, and guiding therapeutic interventions. Research in this area may advance us towards an era of personalized risk prediction and individualized medical therapy. Indeed, since various medications—principally acting on lipid profile and inflammation—may prevent plaque progression or even induce regression, the search for simple techniques makes us able to assess these changes could provide physician a valuable tool for patients management. The present paper, moving beyond coronary stenosis, reviews the features of coronary vulnerable plaques and the ability of CCTA to noninvasive plaque characterization with practical prognostic implication in patient risk stratification. Moreover, current and future therapeutically perspectives are elucidated.

## 2. Definition of Vulnerable Plaque and Features by CCTA

Histologic studies suggest that plaque composition plays a central role in the pathogenesis and clinical consequences of epicardial lesions [41]. Expert consensus points that the morphology, composition, and degree of inflammation of coronary atherosclerotic plaques are more important than the degree of luminal stenosis [42].

If advances in acute coronary syndromes (ACS) are to occur, it is important to recognize their precursor lesions [43]. Most of the ACS are thought to be the result of sudden luminal “thrombosis” that begins from three different pathologies. The most common cause of thrombosis is plaque rupture, followed by plaque erosion. Less commonly dense calcified nodules can penetrate the fibrous cap and cause thrombosis [44–46]. Plaque rupture is the most common cause of coronary thrombosis in both genders: approximately 76% of all fatal coronary thrombi are precipitated by plaque rupture [47, 48]. Consequently, although the term “vulnerable plaque” should be globally reserved for plaques that resemble all three causes of luminal thrombosis, it is usually strictly referred to a rupture-prone plaque. The nonthrombosed lesion that most nearly resembles the acute plaque rupture and then represents its precursor is the thin-cap-fibroatheroma (TCFA) [43].

It has been widely accepted that atherosclerosis is usually a generalized—rather than a focal— process, characterized by a dynamic nature with plaques undergoing biological remodeling and compositional alterations [49]. Autoptic findings from various stages of atherosclerosis have provided a putative sequence of events where lesion progression is not necessarily a process of slow, steady, and indolent accretion [50].

Intimal thickening is observed early in the disease process. The early lesion is composed of smooth muscle cells and is affected by increased macrophage and lipid influx. The next phase is represented by the formation of a necrotic core and the development of a fibrous cap atheroma. The necrotic core contains a certain lipid amount and apoptotic macrophages. Intraplaque hemorrhages are also frequently seen in this entity and lead to further enlargement of the lipid core. A stable fibrous cap may prevent rupture of the lesion. If the fibrous cap loses matrix proteins and smooth muscle cells, a thin cap atheroma can result [51, 52]. The positive remodeling is considered a compensatory outward enlargement of coronary artery accumulating atherosclerosis in its walls [53]. Fibrocalcific plaques might represent an end stage of the atherosclerosis process and can contain extensive calcifications. Because of a stable fibrous cap and lower lipid content, these lesions rarely cause thrombosis but can cause chronic ischemic symptoms because of lumen narrowing [51, 52].

Differently, TCFA are characterized by a large necrotic core, with an overlying thin fibrous cap containing rare smooth muscle cells but numerous infiltrating macrophages [43, 54, 55]. Vessels demonstrating TCFA do not usually show severe lumen narrowing but a positive (expansive) remodeling. Understandably, clinicians aim to detect these plaques before they rupture in order to be able to undertake measures and obtain prevention goals. The search for “vulnerable plaque” is then subject of an intense scientific investigation. Identifying coronary artery lesions prone to future cardiac events and high-risk patients may direct more potent local and systemic approaches for preventive treatments.

Invasive coronary angiography evaluation delineates the vessel lumen with high quality. The additional step of intravascular ultrasound (IVUS), also known as virtual histology, constitutes the current gold standard for plaque evaluation and quantification [56]. Moreover, optical coherence tomography (OCT) provides a higher magnitude of resolution (10 *μ*m) when compared with IVUS (permitting direct visualization of thin cap fibroatheroma) but lacks delineation of the outer vessel boundary due to weaker penetration [57]. Although providing high-resolution images, these techniques are highly expensive and invasive, being used only in conjunction with coronary artery catheterization.

**Figure 1:**
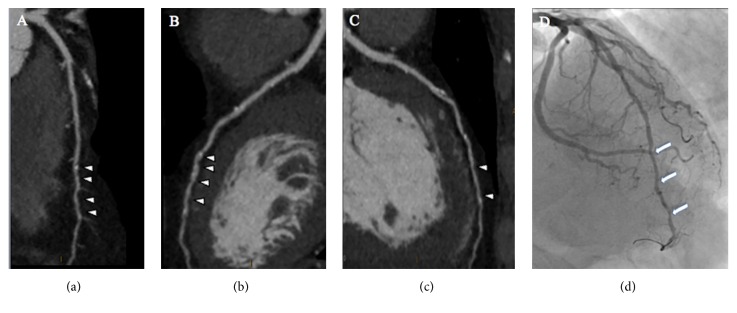
A fifty-two-year-old male patient with familial history of coronary artery disease and inconclusive ECG stress test underwent cardiac computed tomography angiography. Multiplanar reconstruction shows in panels (a), (b), and (c) the presence of severe coronary artery disease at the level of distal left anterior descending artery (arrowhead). Invasive coronary angiography confirmed the diagnosis (panel (c), arrows) and the patient underwent successfully coronary revascularization.

Recently, CCTA has emerged as a promising tool that enables direct visualization of the vascular lumen (with assessment of presence and extent of angiographic stenosis) together with the arterial wall characterization (Figure 1). CCTA focalizes attention on validated measures of plaque vulnerability. There is increasing interest and continuing debate on its potential role as a “noninvasive” method for (1) mapping coronary atherosclerosis, (2) better understanding the adverse features of coronary plaques, and (3) achieving potential benefits in guiding therapeutic interventions [58].

**Figure 2:**
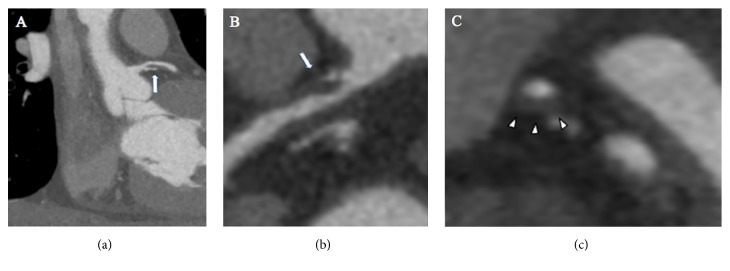
A fifty-three-year old-male patient with history of arterial hypertension and dyslipidemia was admitted at the emergency department for atypical chest pain. Cardiac computed tomography acquired during hospitalization showed in multiplanar reconstruction a soft plaque determining stenosis of 70% in proximal left anterior descending artery ((a) (b) arrow). Cross sectional images showed positive remodeling of a soft plaque ((c) arrowhead).

CCTA imaging has been extensively compared with IVUS and became realty after the demonstration of the existence of a good correlation with virtual histology [59]. Identification of noncalcified plaques (NCP), particularly low-attenuation plaques (LAP) with spotty calcifications (SCPs), positive vessel remodeling (PR), and napkin-ring-like NRS has been considered as important landmarks of plaque vulnerability and instability [60]. Using CCTA, in comparison with grayscale IVUS, calcified versus noncalcified plaque can be quantified on the basis of density cutoff values [61]. Low attenuation suggests high lipid content and has defined for attenuations below 30 Hounsfield Units (HU) [58, 62] (Figure 2). Different HU cut-off limits used in different laboratories presumably have weakened the estimated risk of ACS associated with LAP. Positive remodeling is usually assessed using vessel area (PRI = lesion plaque area/reference area). SCs are scattered calcified nodules within the context of a plaque with a diameter <3 mm. Usually, SCc are well represented on the shoulder of the plaque and are associated to enzyme activity. Finally, the NRS is a thin ring of high attenuation around the plaque along the outer contour of the vessel. This is typically due to the presence of a hypodense deposit of necrotic material in the center of the plaque itself [58, 62]. Importantly, despite ex vivo comparison to histology showed the ability of CCTA to differentiate no calcified, mixed, and calcified plaques [63]. A limitation of commonly used computed tomography (CT) scanners is the relatively poor soft tissue contrast which means difficulty in further subclassification (with possible misclassification) of noncalcified subcomponents (i.e., fibrous versus fatty components) on the only basis of HU attenuation [64, 65]. It has been indeed reported a tendency to overlapping the HU between lipid-rich and fibrous noncalcified plaques. CT technology is, however, rapidly evolving and several solutions are available. In the latest generations of CT devices, faster acquisition speeds have been achieved by faster rotation, larger detectors, and dual source systems. Dual-energy CT can reduce blooming effects that occur near to calcium and iodine and leads to more valid density measurements [66, 67]. The two sources of energy are particularly apt at achieving material decomposition (i.e., differentiation of different tissues), with improved plaque characterization [68, 69]. Moreover, complex image (iterative) reconstructions, recently introduced in commercial systems, seem to be able to improve image quality with regard to noise, resolution, artifacts, and finally diagnostic accuracy [29].

## 3. Prognosis Beyond Degree of Stenosis

Despite advances in preventive approaches and therapies, CAD is one of the main causes of morbidity and mortality in both industrialized and low income to middle-income countries. Sudden cardiac death has been reported to occur in 50% of men and 64% of women without previous cardiovascular symptoms [70]. Coronary stenosis severity is both a powerful but a still now debated predictor of prognosis. A large number of studies have confirmed the long-term prognostic power of CCTA in attributing excellent prognosis to patients (including diabetics) without coronary plaques and intermediate prognosis in patients with nonobstructive lesions. In a long term follow-up, event-free survival rates of symptomatic patients with CT diagnosed CAD decreased proportionally from normal coronary arteries (98.3%) to nonobstructive (95.2%) to obstructive CAD (87.5%) [71]. Similarly, in the very low risk cohort of patients of the CONFIRM registry, followed for a mean of 5 years, Cheruvu et al. reported that the incidence of major adverse cardiovascular events (MACE; all-cause death, nonfatal MI, unstable angina, or late coronary revascularization) increased from 5.6% in those without CAD to 13.24% in those with nonobstructive disease and to 36.28% in those with obstructive CAD (p<0.001) [72].

The novel Coronary Artery Disease-Reporting and Data System (CAD-RADS) scores used to standardize CCTA reporting ranked CAD stenosis severity as 0 (0%), 1 (1% to 24%), 2 (25% to 49%), 3 (50% to 69%), 4A (70% to 99% in 1 to 2 vessels), 4B (70% to 99% in 3 vessels or ≥50% left main), or 5 (100%). It is not surprising that CAD-RADS effectively identify patients at risk for adverse events. Cumulative 5-year event-free survival ranges from 95.2% to 69.3% for CAD-RADS 0 to 5 (p< 0.0001). Higher scores are associated with elevations in event risk (hazard ratio: 2.46 to 6.09; p< 0.0001). Its incorporation into coronary CTA reports may provide a novel opportunity to promote evidence-based care [73]; however, this system, as well as the segment involvement score (SIS), is flawed for several reasons, being probably the main that it oversimplifies prognosis of CAD strictly relating it to the degree of stenosis.

Notably, in a recent substudy of the above mentioned CONFIRM registry, even the presence of a single nonobstructive (1%-49% stenosis) left main plaque in elective CCTA for suspected CAD increased in a 5-year follow-up the risk for composite outcome in women (adjusted hazard ratio, 1.48; p=0.005) but not in men (adjusted hazard ratio, 0.98, p=0.806). This turns out into a nearly 80% higher risk for events than men. This sex-specific prognostic significance, not observed across other patterns (e.g., location or extent) of preclinical coronary plaque, had to be considered since may increase risk stratification efforts [74].

These and similar findings highlight the prognostic importance of both angiographically significant (potentially flow-limiting) and nonobstructive coronary stenosis, as well as the excellent prognosis for patients without evident plaque on CCTA. This means that absence of coronary atherosclerosis on high-resolution CCTA images identifies a patient with an exceptionally low risk of long-term cardiovascular events [75].

Of note, more than two-thirds of acute MI may be due to mild to moderate plaques that did not significantly compromise the coronary lumen before the event [36]. As a consequence, beyond the effective degree of stenosis, other lesion features—reflecting plaque composition—are pivotal determinants of untoward outcomes. The ability of CCTA to assess the entire coronary tree for the presence (present/absent), extent (proximal and/or distal), distribution (per vessel and per segment) of CAD, degree of vessel stenosis (<50% or >50%), and plaque morphology (i.e., calcified, mixed, and no calcified), with further subclassification of plaque subcomponents, makes it a unique non-invasive modality. Starting the first evidence reporting the role of CCTA in improving the prognostic stratification of patients with suspected CAD, there is a growing interest in testing the correlation between the coronary plaque features and the occurrence of MACE [76–78].

In a multicenter study, the presence of a large plaque burden, TCFA, and a small lumen area were independent predictors of future events [56]. Tian et al. demonstrated in 643 patients enrolled in an OCT, IVUS, and angiography study that severe coronary stenosis has a twofold probability to show the features of vulnerable lesions suggesting a potential overlapping between degree of stenosis and plaque characteristics to influence outcome of patients [79]. Undoubtedly, the prevalence of severe coronary stenosis is however significantly lower than that of mild-to-moderate atherosclerotic lesions. Moreover, many of these lesions despite a clinical relevant high plaque burden may be not severely stenotic at ICA.

Ahmadi et al. have showed that survival rate of subjects with nonobstructive CAD decreases significantly with the number of diseased coronary arteries (from single to triple vessels disease, p<0.001) and is significantly affected from the plaque morphology. Death rate increases incrementally from calcified plaque (1.4%) to mixed plaque (3.3%) to no calcified plaque (9.6%). The risk-adjusted hazard ratios of all-cause mortality were 3.2 (95% confidence interval 1.3 to 8.0, p=0.001) for mixed plaques and 7.4 (95% confidence interval 2.7 to 20.1, p=0.0001) for noncalcified plaques compared with calcified plaques. In subjects with mixed or calcified plaques, the death rate also increased with the severity of coronary artery calcium from 1 to 9 to > 400 [30].

High-risk plaque (HRP) features have been also shown to be associated with an increased risk of events even in patients with nonobstructive CAD. In a recently published study it has been shown that the use of an integrated score easily obtained with CCTA (based on the presence of mixed and remodeled atherosclerotic plaques) may improve MACE prediction in symptomatic patients without previous cardiovascular history but at intermediate pretest likelihood of CAD, beyond standard clinical (Diamond & Forrester) and coronary (based on presence and degree of stenosis) scores used in clinical practice [78]. This finding underlines the importance of a comprehensive coronary evaluation even taking into consideration the low prevalence of some high-risk plaque characteristics.

The prognostic value of risk assessment determined on the basis of plaque anatomy alone, however, has been partially disappointing, because of a low positive predictive value [56]. It is indeed worth mentioning that, despite the ability to identify potentially vulnerable plaques with CCTA, there is no clear indication of which and how many plaques with high-risk features will actually rupture and cause events. In the Providing Regional Observations to Study Predictors of Events in the Coronary Tree (PROSPECT) study only 5% of TCFA plaques identified by IVUS caused coronary events [56]. Therefore, the presence of high-risk plaques is probably just a factor in the more complex framework of ACS pathophysiology [80]. The consequences of a plaque disruption depend not only on the composition of the atheroma itself but also on local rheological and hemodynamic phenomena [81]. How plaque composition and local phenomena interact is an important question and several investigators have tried to address it. Moreover, the morphology and underlying activity of individual coronary plaques are heterogeneous and dynamic. Probably, taking into consideration other important pathophysiological principles applied to CCTA imaging, such as plaque inflammation-induced ischemia and the CT-derived fractional flow reserve, it will be conceivable in the next future to further improve the prognostic power of noninvasive coronary evaluation [82].

## 4. CCTA in Asymptomatic Patient

Still evaluating with certainty the role of CCTA in asymptomatic subjects now is not possible and further data are needed to be collected on this topic. Notably, with the technological advance the accuracy of CCTA has constantly improving and, at the same time, possible adverse effects, costs, and radiation exposure reduction are enlarging the indication of the CCTA. Recent recommendations give a criterium of “uncertainty” to the indication of CCTA in asymptomatic patients [Andreini jcm 2016].

The evaluation of asymptomatic patients may sometimes imply a wider evaluation looking for different signs of atherosclerotic involvement of multiple vascular districts. In a cohort of nondiabetic ambulatory subjects, prevalently referred by their physicians for risk-stratification screening, it has been demonstrated that the number of coronary arteries with any amount of disease on CCTA was significantly correlated with increased intima media thickness (IMT) and carotid plaque on vascular ultrasound. CAD was present in most patients with carotid plaque or increased IMT and absent in most patients without carotid plaque or with lower IMT values [83]. Being IMT a well-established marker of subclinical systemic atherosclerotic process and increased global cardiovascular risk beyond traditional system for risk scoring, this relationship supports the concept that an integrated noninvasive approach should be needed [84–86].

Among asymptomatic patients, diabetics represent a particular category in which CCTA could be very useful for prognostic purpose. Two aspects need to be considered. At first, although diabetes mellitus is an important risk factor for future cardiovascular events, some studies suggest that it should not be considered a “coronary risk equivalent” [87]. This consideration is confirmed by studies employing CCTA. Indeed, the absence of coronary atherosclerosis was associated with 100% disease-free survival at follow-up [88]. Second, since the diabetic patient carries a condition of high coronary risk per se [89–91], it is conceivable to postulate that standard risk stratification does not add any additional prognostic information. A recent study supporting this concept has enrolled 517 consecutive asymptomatic patients (63% male, 17.6%diabetics) who underwent CCTA and were evaluated for the prediction of MACE. Over a median follow-up of 4.4 [3.4-5.1] years there were 53 MACE (10%). The authors found that the presence of obstructive CAD and plaque positive remodeling increased MACE prediction as compared to a model based on 10-year-FRS, carotid disease, and coronary calcium scoring in the subgroup of nondiabetic patients. Importantly, the percentage of segments with remodeled plaque was the only predictor of MACE in the subgroup of diabetic subjects [92]. Therefore, CCTA may represent a tool able to make a certain diagnosis of CAD with significant prognostic impact in diabetics. Anyway, since a wide stratification with the use of CCTA of all diabetic patients is not possible for economic reasons, screening patients whit more than 10-year-old diabetes mellitus could be a suitable strategy [93].

## 5. Future Perspectives for Prognosis Improvement

It has been demonstrated that an ischemia-guided revascularization yields improved clinical outcomes in a cost-effective fashion compared with anatomy-guided revascularization alone [38]. As a consequence, in patients with suspected or known disease, noninvasive functional testing should be used as gatekeeper to catheterization. At the time of ICA, the evaluation of fractional flow reserve (FFR) may be instead considered for assessment of the hemodynamic significance of coronary lesion with moderate stenosis (50%-90%). Indeed, the identification of obstructive coronary lesions is only one aspect of the complex relationship between stenosis and ischemia, since there is an increasing awareness on the unreliable relationship between stenosis severity and functional relevance [94].

Even if most CCTA-detected obstructive lesions are confirmed by ICA, lesser than half of those studied with invasive FFR effectively causes ischemia. On the other hand, nonobstructive lesions can be associated with inducible ischemia [95]. Also in this context plaque characterization may help for clinical purpose. Park et al. showed that plaque remodeling, when adjusted for stenosis severity, remained a predictor of ischemia for all degrees of stenosis [96]. Similarly, it has been reported that in moderately stenotic vessels perfusing ischemic territories the prevalence of PR, LAP, and SCs was three to fivefold higher than in vessels without ischemia [97].

The pathogenetic mechanism linking HRP features and inducible ischemia in moderate anatomic stenosis is still not completely clear. It has been postulated that the necrotic core could be responsible for oxidative stress. The resulting local inflammation may compromise the production and bioavailability of the vasodilator nitric oxide and increase the levels of vasoconstrictors such as isoprostanes. The latter along with local endothelial dysfunction could cause a focal “functional stenosis” with inability of the vessel segment containing high-risk plaques to vasodilate adequately during stress [98]. For example, the ongoing presence of endothelial shear stress, which is considered a potent proatherogenic and proinflammatory stimulus, has been associated with a more inflamed and unstable coronary plaque phenotype [56]. Revascularization procedures could be reserved for patients with lower FFR in the presence of obstructive disease on invasive angiography, while high-intensity statin therapy should be prescribed for patients with abnormal FFR in the setting of nonobstructive but high-risk plaques with the aim to obtain plaque stabilization [94].

In this new optic, CCTA with newer applications—due to combination of both plaques characterization and functional evaluation of flow-limiting stenosis in the same examination—seems to represent the Holy Grail for a comprehensive coronary disease assessment [99]. Recently, two methods for the evaluation of the functional relevance of stenosis by cardiac CT have been introduced in the clinical field, stress myocardial computed tomography perfusion (CTP), and fractional flow reserve computed tomography (FFRCT) [24, 82, 100]. Stress CTP demonstrated similar performance to nuclear imaging and additional diagnostic value to CCTA alone as compared to invasive FFR [22]. Software to determine FFR from CCT dataset (FFR-CT) using computational fluid dynamics laws has been recently developed. FFR-CT is derived from routinely anatomic images (acquired at rest only) and subsequent mathematically simulated hyperemia without the need of vasodilator administration.

Gaur et al. showed that plaque tissue characterization and FFR-CT improve the ability to predict inducibility of ischemia in a myocardial territory dependent on a specific coronary lesion compared to mere luminal stenosis assessment [98]. Specific studies have already been designed to investigate whether plaque characterization is a better approach to predict and detect myocardial ischemia compared to current standard of care. Preliminary results from CREDENCE trial are hopefully waited [101].

Moreover, to improve the prognostic power of CCTA, a better clarification of the relationship between plaque burden and cardiac inflammation biomarkers would be very useful [102, 103]. Molecular imaging of plaque activity is also gaining ground and is poised to provide prognostically significant information if the current exciting results are expanded.

## 6. Therapeutic Perspectives

Before CCTA wide spreading, patients without obstructive plaques were often overlooked and, in the absence of inducible ischemia, were included without distinctions in the same group of those without CAD. In fact, among patients with nonobstructive lesions, those with low-risk plaque morphology may be differentiated from those in whom plaque characteristics are associated with an increased risk of future events. Randomized trials have shown that patients undergoing CCTA have significantly reductions in the risk for mortality, revascularizations, and incident MI, probably related to the increased utilization of preventive therapies (i.e., aspirin and statin) among patients with stable chest pain and nonobstructive CAD, as compared to patients who underwent functional provocative test [104, 105].

It is well known that hypocholesterolemic and antiplatelet therapies are considered as some of the most important preventive strategies for coronary artery disease decreasing relative risk of MACE by 20-45% [106, 107].

Reduction in circulating levels of atherogenic lipoproteins has been postulated as one mechanism by which statins exert favorable benefits. However, other pathways beyond cholesterol contribute to CV risk through pleiotropic mechanisms. The statins also reduce intraplaque inflammation, neoangiogenesis, apoptosis, and metalloproteinase activity. These pleiotropic properties, acting together for the plaque stabilization, may contribute to the clinical outcome [108, 109].

Coronary angiography and IVUS techniques for serial examination have demonstrated that statins are able to slow the rate of plaque progression and even to induce a small amount of coronary atherosclerosis regression if target of low-density lipoprotein (LDL) cholesterol levels are achieved. Reduction in LDL cholesterol level to 80 mg/dl by atorvastatin was associated with no increase in coronary plaque burden [110], and more intensive therapy with rosuvastatin to reduce LDL cholesterol to 60 mg/ml results in significant reduction of coronary atherosclerosis [111]. This means a strong relation between cholesterol reduction and changes in atheroma volume.

However, to date, limited data exists to relate the effect of statin use to specifically coronary plaque “features” and morphology beyond stenosis severity [112]. For example, it has been shown that statins increase plaque hyperechogenicity by grey-scale IVUS (independently by plaque volume) and significantly reduce the degree of the fibrofatty intraplaque constituents (conversely increasing intraplaque calcified composition) by virtual histology IVUS [113]. However, IVUS requires an invasive approach and is not suitable for nonischemic patients with nonobstructive plaques (only moderate cardiovascular risk).

Cardiac CT has historically had a role in risk stratification using the Coronary Artery Calcification Score (CAC). CAC is strongly associated with cardiovascular risk. Once coronary calcification is initiated, it follows a predictable pattern of progression, with no consistent evidence of the ability to regress in response to therapy. Although standard CAC score appears to have no role in evaluating therapeutic response or change in atherosclerotic disease over time [40, 114], new CAC scoring approaches discriminating calcium density from volume might provide significant assessment of therapeutic changes, supporting the often asserted (but as yet unvalidated) view that calcification may play a role in plaque stabilization [115].

CCTA is the most promising noninvasive method that has the potential to fully phenotype an individual's coronary artery plaque volume. It has been shown that noncalcified plaques as detected by CCTA represent the component of atherosclerotic plaque that is relevantly influenced by statin therapy and then account for the benefits of therapy [116, 117]. Compared to IVUS, CCTA has undoubtful advantages as noninvasiveness and lower cost. Various studies demonstrated the feasibility of using serial CCTA to assess plaque changes with high intra- and interobserver reproducibility, allowing this method to potentially track atherosclerosis noninvasively. [118]

Inoue et al. in a preliminary study on 32 patients, who underwent CCTA with suspected coronary artery disease, demonstrated that the use of statins—even at a low dosage—resulted in a reduction in plaque quantity and decrease in necrotic core volume. Interestingly, changes in plaque morphology may even occur with relatively less robust changes in the lipid profile and early after initiation of downstream statin treatment [119].

In a recently published multicenter prospective observational study, Li et al. divided patients with baseline mild noncalcified coronary plaque on CCTA according to the statin protocol undertaken [intensive statin therapy (n= 55), moderate statins (n = 85), and no statin (n = 66)]. Their results confirmed that statin can delay progression and even induce plaque regression of mild non-calcified coronary plaque. LAP volume, total plaque volume, and percent plaque volume showed significant regression among intensive statins compared to no statin group. On multivariable model both moderate and intensive statin therapy were independent predictors of plaque regression (although standardized coefficients of the intensive statin was greater than that of the moderate statin: −0.36P < 0.001 vs -0.21 P = 0.004, respectively). Moreover, patients with greater baseline plaque burden and higher basal hyperlipidemia are more likely to benefit from statin therapy. These results could have important implications for disease prevention strategy, suggesting the potential need of stronger statin approach for patients with noncalcified plaque, especially for patients with high risk vulnerable plaque features [117].

The greater benefit from statin therapy even among asymptomatic individuals with higher coronary plaque burden as assessed by CCTA has recently been confirmed also independently from scores for the prediction of 10-year cardiovascular risk [120]. However, despite reducing progression and promoting regression of coronary atherosclerosis, statin therapy just partly addresses residual cardiovascular risk. More than 20% of patients with LDL-C≤70 mg/dL continue to have progression over time in pooled analysis of IVUS studies [40]. This residual risk could potentially be minimized by intensification of lipid-lowering therapy or initiation of non-statin medications, but these approaches are not without drawbacks.

Literature shows that omega-3 fatty acid eicosapentaenoic acid (EPA) has a broad range of beneficial effects on the atherosclerotic pathway, including those on lipids, lipoproteins, inflammation, oxidation, phospholipid membranes, and the atherosclerotic plaque itself [121]. The implications of eicosapent ethyl add-on to statin therapy (in subjects with well-controlled low-density lipoprotein cholesterol levels) for changes in atherosclerotic plaque morphology (plaque burden and/or plaque vulnerability as assessed by CCTA) are currently investigated from ongoing trials that will provide important imaging-derived data [122].

The activation of renin–angiotensin system (RAS) is another important risk factor in atherogenesis. Angiotensin II promotes atherogenesis by stimulating inflammation, oxidative stress, and endothelial dysfunction. In animal models ACE inhibitors and ARBs have been shown to reduce the progression of atherosclerosis [123], and in human study the perindopril has shown to prevent coronary remodeling [124]. Recent studies with CCTA indicate that combination of statins with ACE inhibitor or ARB would be more effective for antiatherosclerotic therapy than statin alone even in patients with CAD, suggesting an inhibitory effect of the combination therapy on vascular remodeling [125].

Also colchicine has been postulated to have beneficial effects on atherosclerosis. In a recently published paper on 80 patients with recent ACS (<1 month) followed for 1 year, colchicine therapy (0.5 mg/day colchicine plus OMT vs OMT alone) was significantly associated with greater reduction in low attenuation plaque volume (p= 0.039) on CCTA, independent of high-dose statin therapy. The improvements in plaque morphology were likely driven by the anti-inflammatory properties, as demonstrated by reductions in high-sensitivity C-reactive protein (hsCRP), rather than changes in lipoproteins. Colchicine could be beneficial as an additional second-lines, add-on, and prevention therapy in patients post-ACS if validated in future studies [126].

Although currently it is not possible to recommend serial scans to monitor the therapeutic efficacy of a medical interventions, the plaque modulation, as a part of risk modification, is a feasible strategy. Direct visualization of the natural course of atherosclerosis, as well as identification of the clinical determinants of plaque progression or regression, holds the potential to shift the paradigm of CAD monitoring among low- to moderate-risk patients with suspected CAD, with aims of offering earlier therapeutic strategies [127]. It is reasonable to accept that a substantial reduction in plaque vulnerability by therapeutic intervention should contribute into plaque stability and in turn decrease cardiovascular event rates. Further studies should be warranted for elucidates this matter.

## 7. Conclusions

Nowadays, primary prevention of major cardiac events needs a strong implementation for ethic and economic reasons. Early identification of CAD, characterization of atherosclerotic process, evaluation of ischemia-related plaque features, and assessment of “vulnerable plaque,” sometimes in the context of “vulnerable patient”, are mandatory endpoints in order to reach this aim. To date, CCTA is the only technique able to approach comprehensively these topics. Moreover, according to the first encouraging literature reports, CCTA could be able to monitor and guide the therapeutic approach which is the ultimate goal of events prediction.
